# Multisystem Critical Illness in Cystic Fibrosis and Pregnancy: Recurrent Enterococcal Urosepsis From Indwelling Bilateral Nephrostomy Tubes

**DOI:** 10.7759/cureus.118030

**Published:** 2026-10-16

**Authors:** Angela Chen, Shelena Ali-Bynom

**Affiliations:** 1 Osteopathic Medicine, Nova Southeastern University Dr. Kiran C. Patel College of Osteopathic Medicine, Clearwater, USA; 2 Obstetrics and Gynecology, Lakeland Regional Medical Center, Lakeland, USA

**Keywords:** cftr modulators, cystic fibrosis, nephrostomy, pregnancy, urosepsis

## Abstract

We report a 22-year-old Gravida 1, Parity 0 (G1P0) woman with cystic fibrosis (CF) maintained on elexacaftor/tezacaftor/ivacaftor (ETI; Trikafta) who sustained four hospitalizations for recurrent extended-spectrum beta-lactamase (ESBL)-producing Klebsiella pneumoniae urosepsis in the four months preceding this admission, ultimately requiring bilateral percutaneous nephrostomy tube placement. Five days after completing a 14-day course of intravenous meropenem delivered via peripherally inserted central catheter (PICC), she presented at 27 weeks and six days of gestation with fever and left-sided flank pain. On hospital day two, she developed hemodynamic compromise consistent with severe sepsis. Blood and urine cultures confirmed *Enterococcus faecalis* bacteremia. The emergence of *Enterococcus faecalis* raised concern for antibiotic-driven enterococcal selection pressure from repeated carbapenem exposure. The hospitalization was complicated by acute anemia requiring transfusion of two units of packed red blood cells (PRBC), with the lowest recorded value of hemoglobin being 6.7 g/dL (normal 11.6-15.0 g/dL). Procalcitonin, a marker of bacterial infection severity, was elevated at 3.64 ng/mL (normal ≤0.15 ng/mL). Serum lactate was measured serially throughout the intensive care unit (ICU) stay and remained within normal limits, with a peak value of 1.5 mmol/L (normal 0.5-2.2 mmol/L). By the Sepsis-3 consensus definition, a serum lactate above 2.0 mmol/L in conjunction with vasopressor requirement is needed to meet the formal diagnosis of septic shock; this patient did not meet those criteria at any measured time point. Fetal echocardiography revealed atrial bigeminy with frequent premature atrial contractions (PACs), which resolved with maternal hemodynamic stabilization. Despite the severity of antepartum illness, the patient delivered a healthy male infant via spontaneous vaginal delivery at term, with Apgar scores of 8 and 9 at one and five minutes and a birth weight of approximately 8 lbs. She breastfed exclusively postpartum while continuing ETI. The establishment of a structured four-week nephrostomy tube exchange schedule under a perioperative antibiotic umbrella represents a structural source-control strategy with broad applicability to similar high-risk patients.

## Introduction

Historically, pregnancy in women with cystic fibrosis (CF) was considered rare and exceptionally high-risk. The advent of CF transmembrane conductance regulator (CFTR) modulator therapy has significantly shifted the reproductive landscape, leading to a rise in conception rates among women with CF. Despite these advances, CF pregnancies remain clinically complex: 31% require hospitalization for infective pulmonary exacerbations [1], and women with CF face substantially higher odds of maternal death compared with those without CF [2]. Between 2000 and 2019, the prevalence of CF at delivery rose from 2.1 to 10.4 per 100,000 deliveries, and the proportion presenting with additional chronic conditions increased from 18% to 37% [2].

Indwelling nephrostomy tubes during pregnancy introduce additional morbidity, most notably urosepsis, as progesterone-induced ureteral dilation and compression by the gravid uterus promote urinary stasis and ascending infection [3,4]. The convergence of CF, pregnancy, and recurrent nephrostomy-related urosepsis constitutes an exceedingly rare clinical triad. We present this case to describe the diagnostic and management strategies employed across infectious disease, maternal-fetal medicine, critical care, and urology, to propose a source-control framework for pregnant patients with indwelling urologic hardware and recurrent gram-positive bacteremia, and to highlight the limitations of serum lactate as a sepsis severity marker in the setting of augmented renal clearance physiology inherent to CF and pregnancy. We hope this report provides a practical clinical reference for multidisciplinary teams encountering similarly complex presentations at the intersection of CF, pregnancy, and critical illness.

## Case presentation

Background and antecedent hospitalizations

A 22-year-old Gravida 1, Parity 0 (G1P0) female was diagnosed with cystic fibrosis (CF) and managed on elexacaftor/tezacaftor/ivacaftor (ETI; Trikafta) and pancrelipase. She had a history of recurrent nephrolithiasis and multidrug-resistant (MDR) and extended-spectrum beta-lactamase (ESBL)-producing Klebsiella pneumoniae urinary tract infections. She conceived spontaneously and was followed in a high-risk obstetric clinic.

Her antepartum course was complicated by recurrent ascending urinary tract infections requiring percutaneous source control. A left percutaneous nephrostomy tube was placed at approximately 14 weeks of gestation for ESBL Klebsiella pneumoniae pyelonephritis with ureteral obstruction. She was subsequently rehospitalized for urosepsis treated with intravenous ertapenem. At approximately 24 weeks of gestation, she was admitted again for urosepsis with concurrent pulmonary involvement, during which a right nephrostomy tube was placed, and the left nephrostomy tube was exchanged.

She completed a 14-day course of intravenous meropenem delivered via a peripherally inserted central catheter (PICC). The meropenem course was the primary antibiotic treatment for the preceding septic admission. Once completed, the PICC line was removed five days before this presentation. Weekly intramuscular (IM) ertapenem suppressive therapy had been planned but not yet initiated. A clinical timeline illustrating the full antepartum and postpartum course is presented in Figure 1.

**Figure 1:**
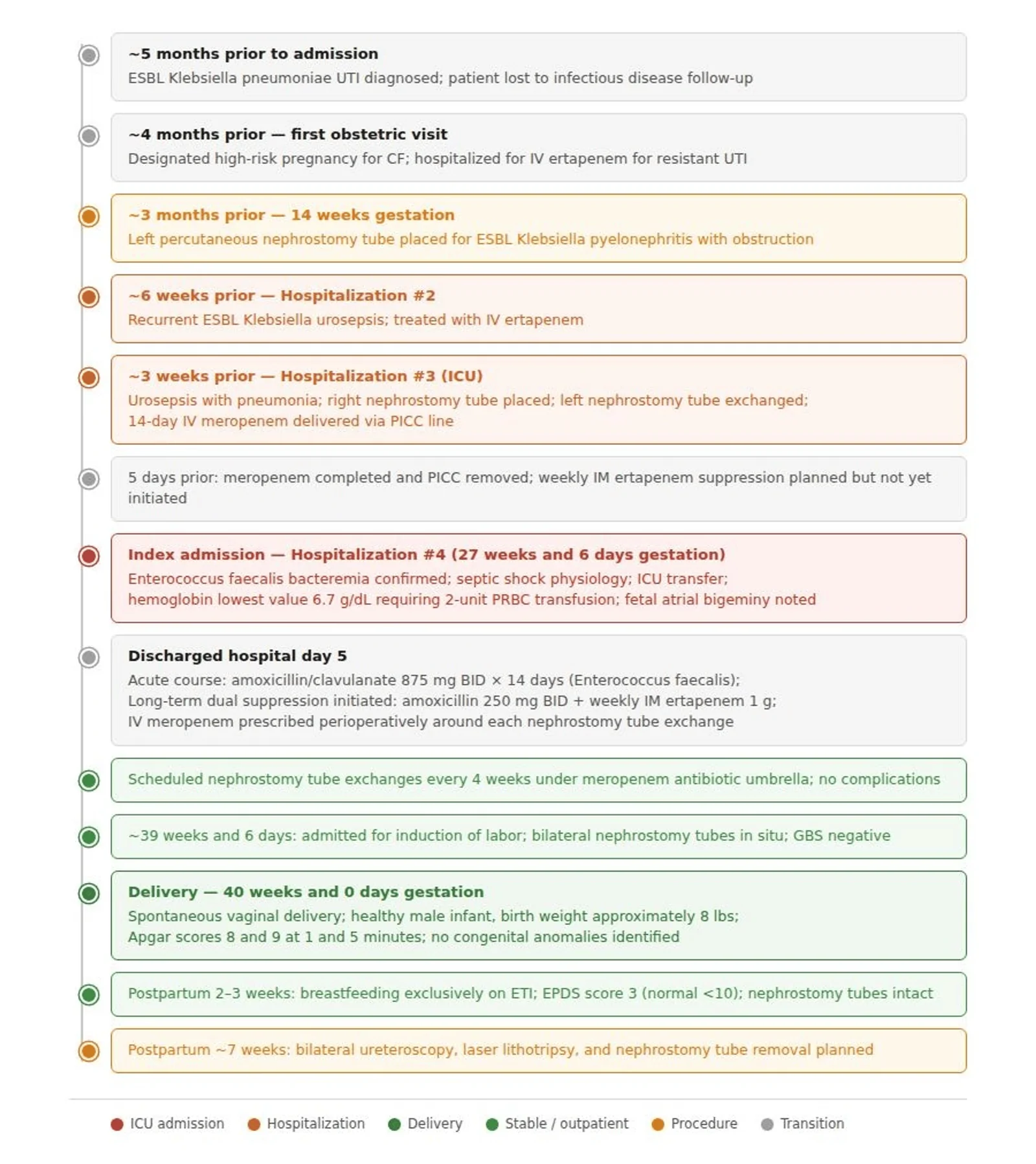
Clinical timeline-antecedent course through postpartum outcome Clinical timeline spanning the antepartum and postpartum course, from initial diagnosis of Klebsiella urinary tract infection through bilateral nephrostomy tube placement, four hospitalizations, index septic admission, term delivery, and postpartum urologic follow-up. UTI: urinary tract infection; CF: cystic fibrosis; ESBL: extended-spectrum beta-lactamase; ICU: intensive care unit; IV: intravenous; PICC: peripherally inserted central catheter; PRBC: packed red blood cells; BID: twice a day; IM: intramuscular injection; GBS: Group B Streptococcus; ETI: elexacaftor/tezacaftor/ivacaftor; EPDS: Edinburgh Postnatal Depression Scale.

Index presentation

The patient presented to the obstetric emergency department at 27 weeks and six days of gestation with new-onset fever and left flank pain. The day prior, she had noticed a small amount of blood in the left nephrostomy tube collection bag, which temporarily improved after flushing the tube. Pain and fever then returned, prompting her to seek care.

On arrival, her oral temperature was 37.3°C (normal ≤37.5°C), heart rate was 121 bpm (normal 60-100 bpm), and blood pressure was 111/57 mmHg. Peripheral oxygen saturation (SpO₂) was 94% on room air (normal ≥95%). The left nephrostomy tube insertion site had mild surrounding erythema with crusting, while the right site appeared normal. Continuous external fetal monitoring demonstrated a fetal heart rate of 160 bpm with moderate variability and no decelerations, which was reassuring for gestational age [5]. Empiric intravenous ertapenem was initiated based on her known ESBL history.

Decompensation and diagnostic workup

On hospital day two, the patient's clinical status deteriorated rapidly. She developed persistent fever (peak oral temperature 39.1°C; axillary peak 39.7°C on hospital day three), tachycardia (heart rate 142-152 bpm), and tachypnea (respiratory rate 46-60 breaths per minute).

Her systolic blood pressure (SBP) fell to a low of 69 mmHg despite receiving 2.5 liters of intravenous crystalloid resuscitation. She was transferred to the intensive care unit (ICU) and placed on high-flow nasal cannula (HFNC) oxygen, achieving SpO₂ of 96%. An intensivist was involved in her care throughout the ICU stay. Arterial blood gas (ABG) measurement was not documented in the available chart records, which represents a limitation of this case report. Objective ventilatory data would have been valuable given respiratory rates as high as 60 breaths per minute. Intravenous vancomycin was added for empiric Gram-positive coverage.

Computed tomography (CT) angiography of the chest excluded pulmonary embolism and showed stable subsegmental consolidations with stable scattered foci of cystic bronchiectasis, with no new acute findings beyond baseline CF features. The CT angiography is presented in Figure 2. Renal ultrasonography showed grade one right-sided hydronephrosis with an 11-mm non-obstructive calculus in the right renal pelvis and punctate non-obstructive calculi bilaterally. The renal ultrasound findings are presented in Figures 3, 4, and the coronal CT abdomen and pelvis is presented in Figure 5, demonstrating the right nephrostomy tube in situ (arrow A) and the gravid uterus (arrow B).

**Figure 2:**
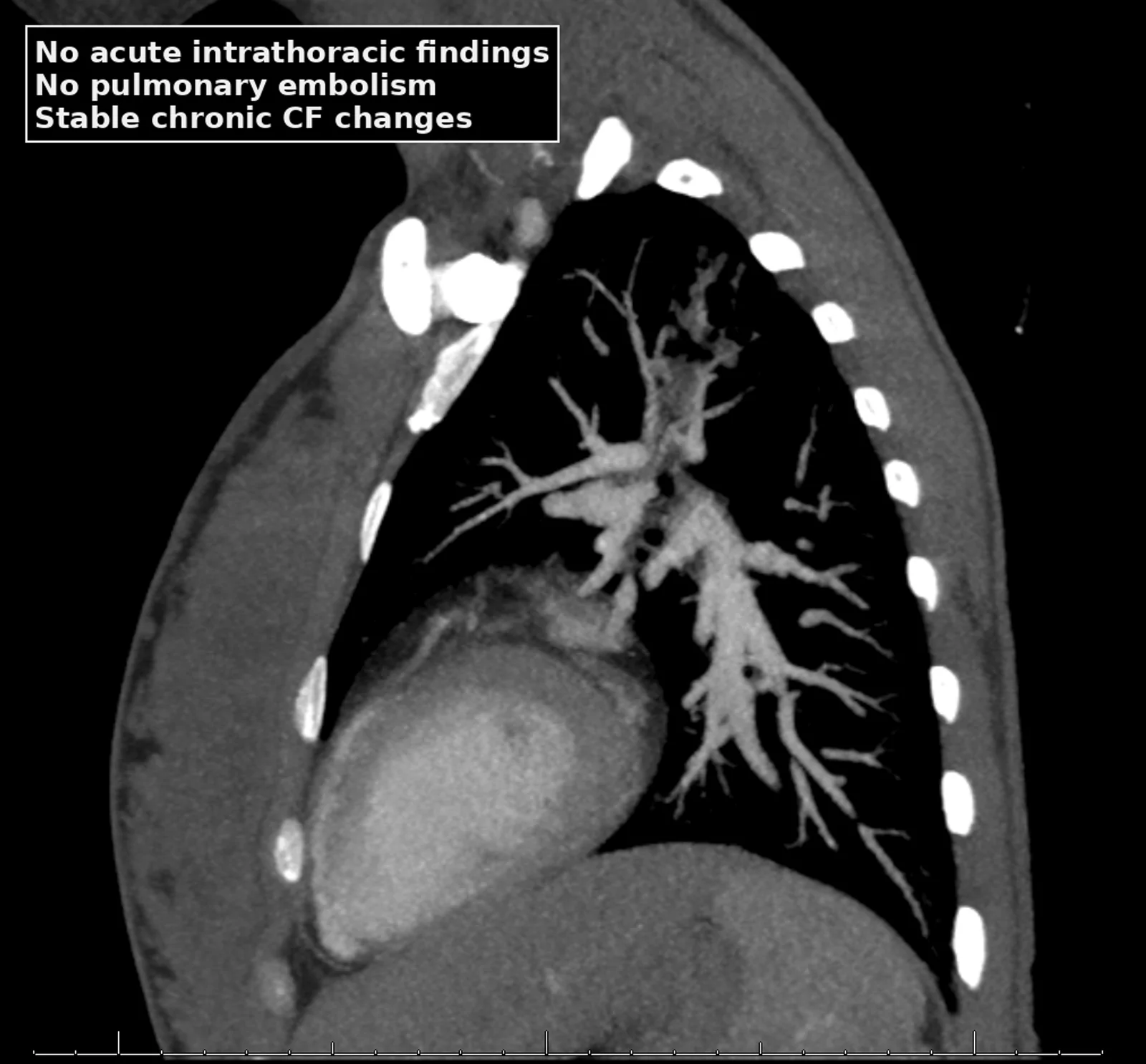
CT angiography of the chest MIP reconstruction from CT pulmonary angiography. The arrow indicates the central pulmonary vasculature. No intraluminal filling defect was identified, excluding PE. The study demonstrated stable chronic cystic fibrosis parenchymal changes with no new acute intrathoracic findings. CF: cystic fibrosis; CT: computed tomography; MIP: maximum intensity projection; PE: pulmonary embolism.

**Figure 3:**
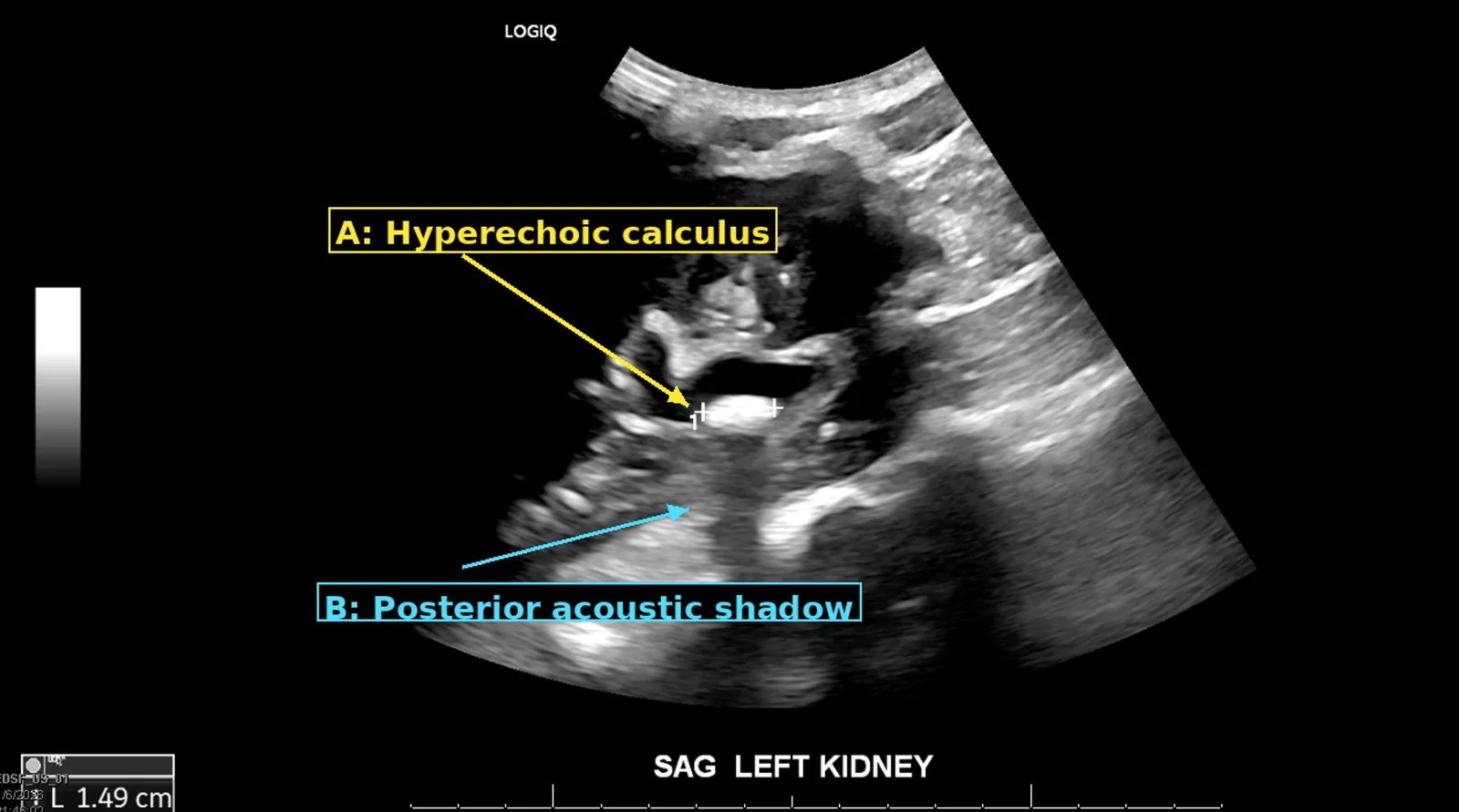
Sagittal ultrasound of the left kidney Arrow A (yellow) indicates a hyperechoic focus consistent with a renal calculus, measuring 1.49 cm on calipers. Arrow B (cyan) indicates the posterior acoustic shadow distal to the calculus, a characteristic sonographic feature of urolithiasis.

**Figure 4:**
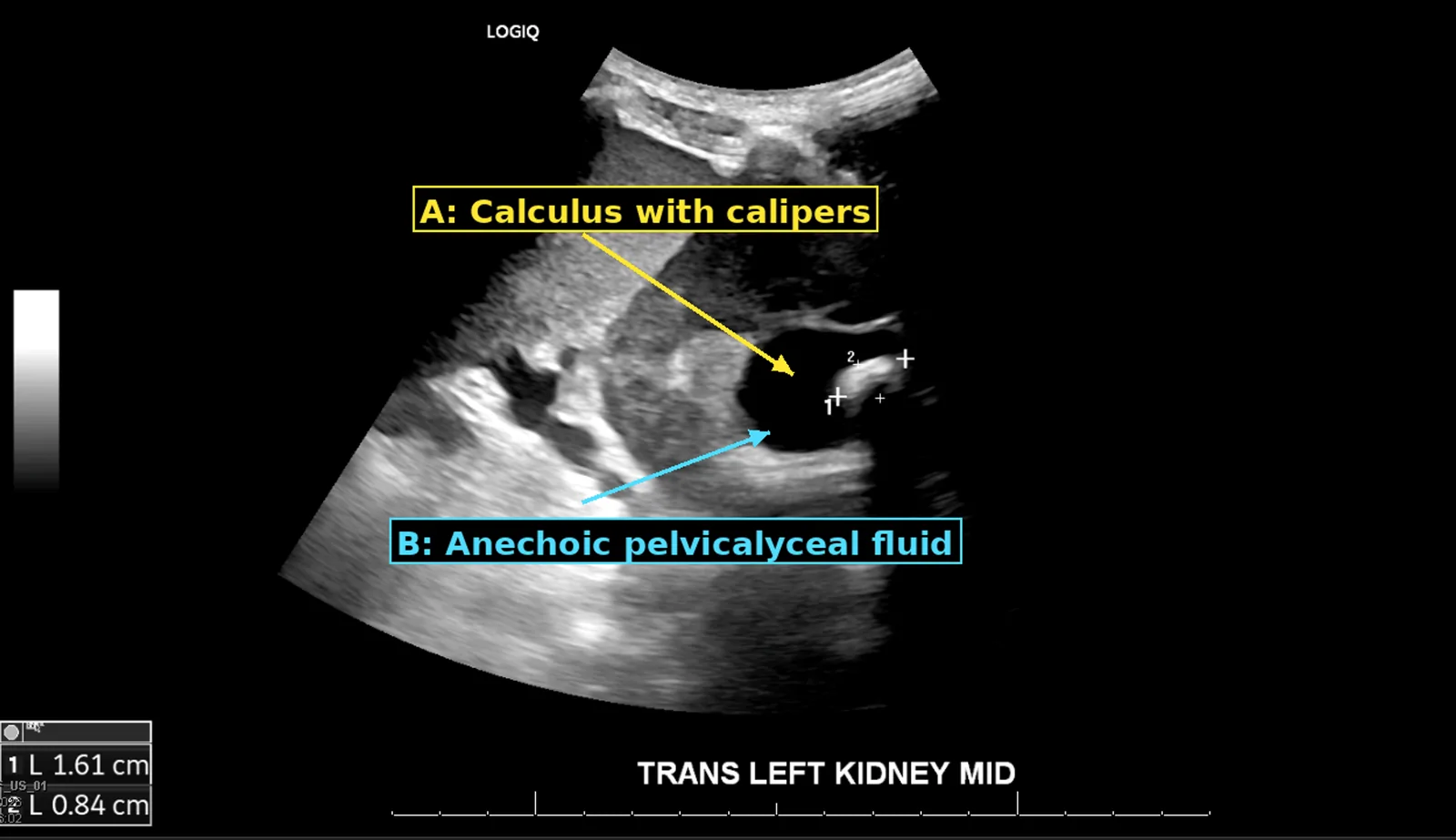
Renal ultrasonography - transverse view, left kidney Transverse ultrasound of the left kidney mid-pole. Arrow A (yellow) indicates the calculus with sonographic calipers measuring 1.61 × 0.84 cm in the transverse dimension. Arrow B (cyan) indicates the anechoic pelvicalyceal fluid consistent with mild hydronephrosis.

**Figure 5:**
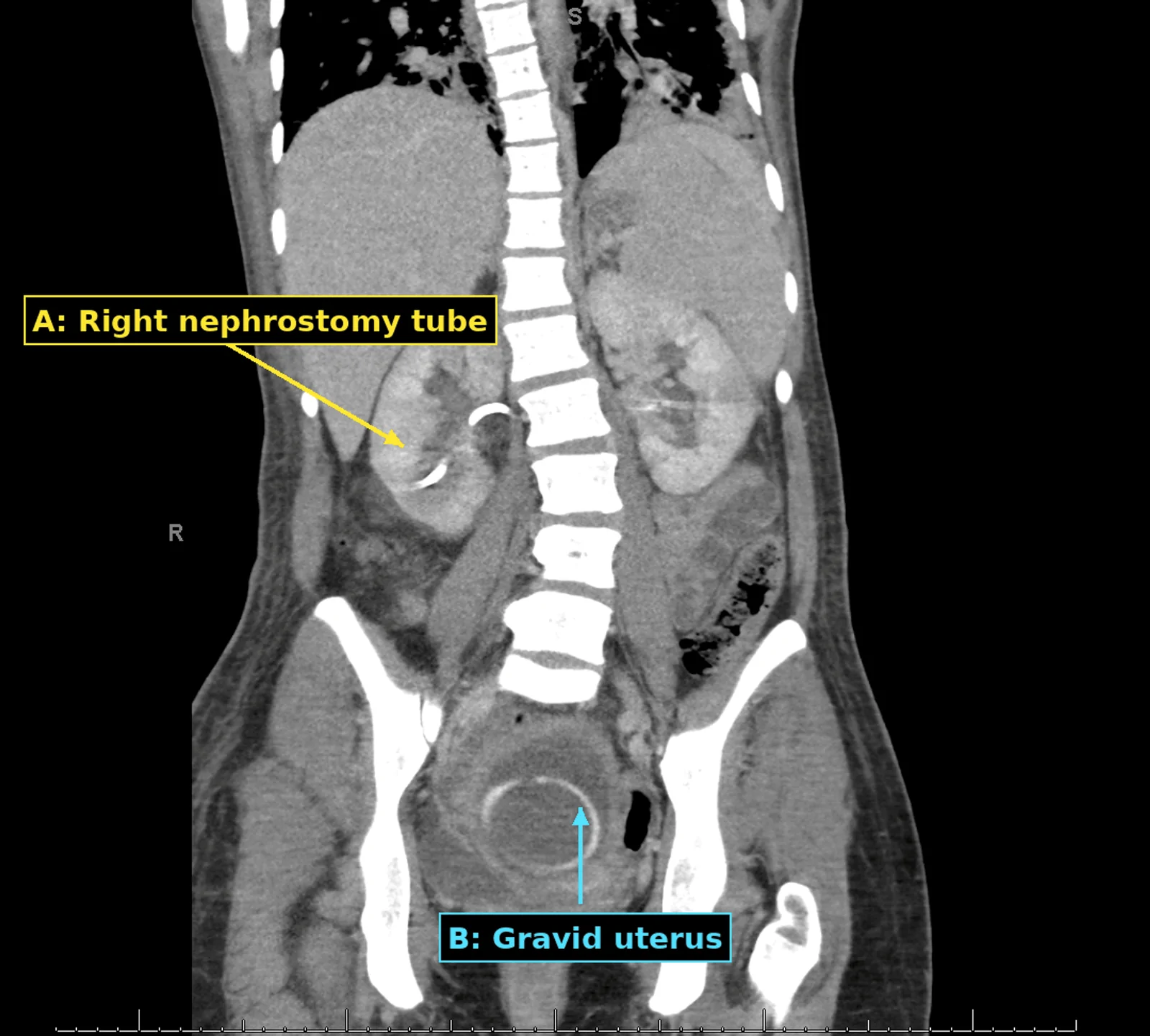
CT of the abdomen and pelvis - coronal view Coronal CT of the abdomen and pelvis (status post right nephrostomy tube placement). Arrow A (yellow) indicates the right nephrostomy tube in the right renal pelvis. Arrow B (cyan) indicates the gravid uterus occupying the pelvis, consistent with the third trimester of pregnancy. Radiologic orientation: patient's right is on viewer's left (R marker visible). CT: computed tomography.

Blood cultures drawn on admission and urine cultures from both nephrostomy tubes grew *Enterococcus faecalis*, sensitive to ampicillin and vancomycin. Procalcitonin (PCT), a validated biomarker that rises in response to bacterial infection and correlates with sepsis severity [6], was measured on hospital day four and returned elevated at 3.64 ng/mL (normal ≤0.15 ng/mL), confirming significant bacterial infection. Serum lactate was measured at multiple time points during the ICU stay and remained within normal limits throughout (peak 1.5 mmol/L; normal 0.5-2.2 mmol/L). Coagulation studies on hospital day two showed a mildly elevated prothrombin time (PT 14.0 seconds; normal 10.2-12.9 seconds) and international normalized ratio (INR) 1.2; normal 0.8-1.1, with a normal activated partial thromboplastin time (APTT) of 27.8 seconds; normal 25.0-36.5 seconds. Fibrinogen and D-dimer were not obtained during this admission. Antibiotic coverage was narrowed to ampicillin/sulbactam 3 g intravenous every six hours on hospital day three after culture sensitivities returned. Serial vital signs and laboratory values are summarized in Table 1.

**Table 1:** Vital sign and laboratory trends during index hospitalization HD: hospital day; HR: heart rate; SBP: systolic blood pressure; RR: respiratory rate; SpO₂: peripheral oxygen saturation; RA: room air; HFNC: high-flow nasal cannula; WBC: white blood cell count; Hgb: hemoglobin; PT: prothrombin time; APTT: activated partial thromboplastin time; INR: international normalized ratio; ‡ Lowest recorded value; indication for transfusion; § Post-transfusion value; † Reference range reflects non-pregnant adult; serum creatinine typically decreases approximately 30% in pregnancy due to increased glomerular filtration rate. Arterial blood gas was not documented in available chart records.

Parameter	HD 1	HD 2	HD 3	HD 4	Discharge	Reference Range
Temp Max (°C)	38.4	39.1	39.7	36.9	36.8	≤37.5
HR (bpm)	121	142–152	134	108	100	60–100
SBP nadir (mmHg)	111	69	104	113	112	≥90
RR (bpm)	16	46–60	38	24	20	12–20
SpO₂ (%)	94 RA	96 HFNC	94 HFNC	100	99	≥95
WBC (K/µL)	12.98	11.66	14.41	10.99	7.47	3.4–9.6
Hgb (g/dL)	11.8	10.5	8.4	6.7 ‡	9.2 §	11.6–15.0
Platelets (K/µL)	223	197	135	113	118	157–371
Creatinine (mg/dL)	0.44	0.44	0.44	0.41	0.39	0.51–0.95 †
Lactate (mmol/L)	—	0.8–1.4	1.1	1.5	—	0.5–2.2
Procalcitonin (ng/mL)	—	—	—	3.64	—	≤0.15
PT (sec)	—	14	—	12.7	—	10.2–12.9
APTT (sec)	—	27.8	—	—	—	25.0–36.5
INR	—	1.2	—	1.1	—	0.8–1.1
Albumin (g/dL)	3.52	3.04	2.56	2.58	—	3.97–4.94

Hospital course

The hospitalization was complicated by acute anemia. Hemoglobin (Hgb) fell from 11.8 g/dL on admission to a lowest recorded value of 6.7 g/dL on hospital day four (normal 11.6-15.0 g/dL). Platelet count fell to a lowest point of 113 K/µL (normal 157-371 K/µL), and albumin declined to 2.56 g/dL (normal 3.97-4.94 g/dL). The patient received two units of packed red blood cells (PRBC), with an appropriate response, rising to 9.2 g/dL after transfusion.

The rapid hemoglobin decline was likely multifactorial, including hemodilution from crystalloid resuscitation, sepsis-associated suppression of erythropoiesis, and ongoing low-grade hematuria from the nephrostomy tubes. The diagnostic implications of this gap are discussed in the Acute Anemia section of the Discussion.

Fetal echocardiography on hospital day three demonstrated normal segmental cardiac anatomy, with an underlying atrial rhythm at 150-160 bpm and frequent premature atrial contractions (PACs) in a pattern of atrial bigeminy. No fetal hydrops or sustained tachyarrhythmias were observed. The PACs resolved as maternal hemodynamics stabilized.

Following ICU stabilization, urology identified early encrustation of both nephrostomy tubes as the probable driver of recurrent bacteremia and recommended deferring the nephrostomy tube exchange until the acute infection had fully resolved. A post-acute exchange interval of every four weeks under a perioperative intravenous antibiotic umbrella was then established.

On hospital day five, the patient was discharged on oral amoxicillin/clavulanate 875 mg twice daily to complete a 14-day acute course targeting *Enterococcus faecalis*. Long-term dual suppressive therapy was initiated: oral amoxicillin 250 mg twice daily for ongoing *Enterococcus *coverage and weekly intramuscular (IM) ertapenem 1 g for ESBL Klebsiella coverage, based on her documented prior bacteriology. Intravenous meropenem was prescribed for the day before and the day after each future nephrostomy tube exchange.

Antepartum follow-up and delivery

Subsequent nephrostomy tube exchanges were performed on the four-week schedule under the meropenem umbrella without complication. Growth ultrasonography at approximately 28 weeks showed estimated fetal weight at the 62nd percentile for gestational age (normal: 10th-90th percentile). Antenatal testing was initiated and remained reassuring. A one-hour glucose challenge test (GCT) result was 116 mg/dL, within normal limits (normal: <140 mg/dL). ETI and amoxicillin suppression were continued throughout the antepartum course.

At approximately 39 weeks and six days of gestation, the patient was admitted for induction of labor (IOL), indicated by her CF-complicated obstetric course. Both nephrostomy tubes remained in place and were draining well. She was negative for Group B Streptococcus (GBS), a vaginal and rectal bacterial colonization screened at 36-38 weeks because GBS can cause serious neonatal infection if transmitted during delivery. She delivered a healthy male infant via spontaneous vaginal delivery at 40 weeks and 0 days, with Apgar scores of 8 and 9 at one and five minutes and a birth weight of approximately 8 lbs. Bilateral labial lacerations were repaired, and the immediate postpartum course was uneventful.

Postpartum course

At an early postpartum visit, the patient reported that she was breastfeeding exclusively and feeling well. She was screened using the Edinburgh Postnatal Depression Scale (EPDS), a validated 10-item questionnaire used to identify postpartum depression [7]; scores of 10 or above suggest possible depression. Her score of 3 was well below this threshold, indicating no significant depressive symptoms at that time. She remained on Trikafta, amoxicillin suppression, prenatal vitamins, and Vitamin D supplementation. Bilateral nephrostomy tubes were draining clear urine without signs of infection.

Postpartum imaging identified residual distal right ureteral calculi and a left renal pelvic stone. Urology planned bilateral ureteroscopy with laser lithotripsy and nephrostomy tube removal, with oral antibiotics to continue for one week following the procedure. The infant had no congenital anomalies identified at initial assessment.

## Discussion

The three-way physiologic tension

Management of severe sepsis in a pregnant patient with CF requires navigating three competing physiologic demands: the volume-expanded, vasodilated hemodynamics of pregnancy; the chronic suppurative and restrictive-obstructive pulmonary pathology of CF; and the resuscitation requirements of distributive shock [5,7]. The Society for Maternal-Fetal Medicine (SMFM) Consult Series #67 recommends limiting crystalloid resuscitation to one to two liters of balanced crystalloid. This is especially important in CF, where lower colloid oncotic pressure and impaired mucociliary clearance amplify the risk of pulmonary edema [5]. In this case, approximately 2.5 liters of crystalloid were administered prior to ICU transfer without overt pulmonary edema, though HFNC oxygen support was transiently required. Norepinephrine is the recommended first-line vasopressor (GRADE 1C) to maintain mean arterial pressure (MAP) and uteroplacental perfusion, with intravenous hydrocortisone 200 mg/day reserved for vasopressor-refractory shock [5,8]. In this patient, vasopressors were not required, and hydrocortisone was not initiated; hemodynamic recovery was achieved with fluid resuscitation, source control, and targeted antimicrobial therapy alone. The compensated respiratory alkalosis of normal pregnancy establishes a baseline partial pressure of carbon dioxide (PaCO₂) of 28-32 mmHg. In a pregnant CF patient, a PaCO₂ of 40 mmHg should therefore be recognized as a sign of impending respiratory failure, not a normal value [7,9].

Sepsis definition and the limits of lactate

The Sepsis-3 consensus defines septic shock as sepsis requiring vasopressor support to maintain mean arterial pressure ≥65 mmHg with a serum lactate above 2.0 mmol/L despite adequate volume resuscitation [10]. By these strict criteria, this patient did not meet the definition of septic shock at any time point when lactate was measured: her peak lactate was 1.5 mmol/L, and vasopressors were not ultimately required. However, her clinical picture was consistent with severe sepsis: fever to 39.7°C, tachycardia to 152 bpm, tachypnea to 60 breaths per minute, and a systolic blood pressure nadir of 69 mmHg [6,10]. Clinicians should interpret lactate values alongside the full clinical picture. In a patient with markedly augmented renal clearance, as evidenced by a creatinine persistently measuring 0.39-0.44 mg/dL (well below the non-pregnant reference range of 0.51-0.95 mg/dL), a normal lactate may reflect either preserved tissue perfusion or accelerated lactate clearance [6,11].

Source control: from acute deferral to structural prevention

Rapid identification and control of the infectious source is the most critical management priority in maternal sepsis [5,8]. While ureteral stents are associated with fewer urinary tract infections [adjusted odds ratio (aOR 0.52)] and fewer repeat procedures (aOR 0.70) compared to nephrostomy tubes in pregnancy [3], this patient's obstructive calculi required nephrostomy tube placement. Ureteral stenting was considered but deemed less suitable given the stone burden and ureteral anatomy; nephrostomy tube placement was pursued following shared decision-making with urology. Approximately 80% of pregnant patients with nephrostomy tubes require intervention before the standard six-to-eight-week exchange interval [3]. The five-day interval between PICC removal and the planned initiation of weekly intramuscular ertapenem suppressive therapy may have contributed to this septic presentation. In a patient with documented ESBL colonization and indwelling bilateral nephrostomy tubes, any gap in suppressive antibiotic coverage represents a plausible window of vulnerability, though causality cannot be established from a single case. This observation nonetheless argues for initiating suppressive therapy before or concurrent with PICC removal rather than deferring it afterward. Of note, the patient had been lost to infectious disease follow-up in the five months preceding this admission; the specific reason was not documented in the available medical records. Regardless of etiology - whether access-related, logistical, or patient-driven - this lapse likely compounded the risk of undetected colonization progression and underscores the importance of proactive, team-initiated outreach to maintain continuous infectious disease co-management in high-risk pregnant patients with chronic urologic hardware. This case illustrates two complementary source-control principles. During acute bacteremia, the urology team appropriately deferred nephrostomy tube manipulation to avoid worsening seeding. Once the acute illness resolved, a structured prophylactic schedule was instituted: four-week exchanges under a perioperative meropenem umbrella, representing a shift from reactive to structural source control [5].

Antimicrobial stewardship: the carbapenem-to-*Enterococcus* shift

The microbiologic progression from ESBL Klebsiella pneumoniae to *Enterococcus faecalis* across four hospitalizations warrants discussion. Carbapenems lack reliable enterococcal activity. Repeated carbapenem courses can promote the selective overgrowth of intrinsically resistant enterococcal organisms, particularly in the setting of indwelling urologic hardware [6,11]. This phenomenon is a recognized risk of prolonged broad-spectrum antibiotic exposure, and the temporal pattern in this case is consistent with antibiotic-driven microbiome disruption [6,12].

Empiric ertapenem appropriately targeted the known ESBL history on this admission, and vancomycin was appropriately added upon decompensation for Gram-positive coverage. De-escalation to ampicillin/sulbactam following identification of *Enterococcus faecalis* is consistent with antimicrobial stewardship principles, which recommend narrowing therapy to the most targeted effective agent once a pathogen and its sensitivities are identified [11,12]. The dual suppression regimen at discharge, with amoxicillin targeting *Enterococcus* and weekly intramuscular ertapenem targeting ESBL Klebsiella, was specifically designed to provide coverage against both organisms documented in her chronic colonization profile.

Pharmacokinetic considerations

A critical challenge in this population is the pharmacokinetic double alteration: both CF and pregnancy independently increase total body clearance and the volume of distribution for beta-lactam antibiotics [8]. Population pharmacokinetic modeling in CF patients has shown that standard dosing regimens frequently fail to achieve target minimum inhibitory concentration (MIC) attainment. Prolonged or continuous infusion strategies improve the probability of pharmacokinetic/pharmacodynamic target attainment several-fold compared to standard intermittent dosing [12,13]. International consensus guidelines now endorse prolonged-infusion beta-lactam strategies for adults with severe infections [13]. However, no prospective clinical outcome data exist specifically for pregnant CF patients, and these strategies remain hypothesis-generating in this population. The patient's persistently low serum creatinine values reinforced concern for accelerated antibiotic elimination, and therapeutic drug monitoring (TDM) warrants consideration in future admissions [14].

CFTR modulator therapy: continuation throughout pregnancy and postpartum

The patient remained on ETI throughout her antepartum course, all ICU-level hospitalizations, delivery, and into the postpartum period while breastfeeding exclusively. A French nationwide cohort study of 590 CF pregnancies found no significant difference in major birth defects between ETI-exposed and unexposed groups (3.38% vs 4.66%; p = 0.72) [15]. The maternal cost of withdrawal is clinically significant: five of six women who discontinued ETI due to fetal safety concerns required reinitiation because of rapid clinical deterioration [16]. The healthy term delivery with normal Apgar scores in this case is consistent with current reassuring ETI safety data, and contributes to the growing literature supporting continued ETI use during pregnancy [15-17]. The ongoing Modulator Approaches in cYstic Fibrosis: Long-tErm Outcomes With Reproductive Safety (MAYFLOWERS trial; NCT04828382) is a prospective observational study specifically designed to evaluate the safety of CFTR modulator therapy in pregnancy and lactation [18]. The Trikafta prescribing information (section 5.7) warns of non-congenital lens opacities in pediatric patients on ivacaftor-containing regimens [19]; routine infant ophthalmologic screening is recommended postpartum regardless of breastfeeding status.

Fetal and neonatal outcomes

The PACs observed on fetal echocardiography on hospital day three resolved with maternal hemodynamic stabilization. Fetal PACs are common, occurring in approximately 1-2% of pregnancies, and are most often benign and self-limiting [5]. The resolution of PACs with maternal stabilization supports a catecholamine-mediated mechanism. However, this observation is hypothesis-generating from a single case and requires prospective validation before it can be adopted as a clinical monitoring tool [5,9]. Delivery was not pursued for the sole indication of sepsis, consistent with SMFM guidance that delivery should be driven by maternal-fetal condition rather than infection alone [5].

Acute anemia: mechanistic considerations

The hemoglobin decline from 11.8 to 6.7 g/dL over four hospital days was likely multifactorial. Contributing factors included hemodilution from approximately 5 liters of net positive fluid balance over 48 hours, sepsis-associated erythropoietic suppression, and ongoing low-grade hematuria from the nephrostomy tubes [6]. The coagulation profile did not meet criteria for disseminated intravascular coagulation (DIC): prothrombin time (PT) was mildly elevated at 14.0 seconds, activated partial thromboplastin time (APTT) was normal at 27.8 seconds, and the platelet count reached a lowest value of 113 K/µL. However, fibrinogen and D-dimer were not obtained, which represents a diagnostic gap. These tests should be included in the routine sepsis workup of pregnant patients with acute anemia, as the physiologically elevated fibrinogen of late pregnancy can mask early DIC [6,11].

Postpartum care: airway clearance, lactation, and urologic planning

Adequate pain management following delivery is essential in CF patients to enable effective airway clearance and chest physiotherapy, which should be resumed as early as possible postpartum [12]. The European Respiratory Society (ERS) and Thoracic Society of Australia and New Zealand (TSANZ) Task Force on the management of reproduction and pregnancy in women with airway diseases specifically recommends early postpartum reestablishment of airway clearance regimens [12]. Spontaneous vaginal delivery in this patient allowed earlier ambulation and chest physiotherapy compared to cesarean delivery. The patient's Edinburgh Postnatal Depression Scale (EPDS) score of 3, well below the screening threshold of 10 [7], was reassuring; however, clinicians should monitor for postnatal depression in this population, as data on its prevalence in CF pregnancies are limited [1]. The patient was breastfeeding exclusively while continuing ETI. The Trikafta prescribing information (section 5.7) recommends ophthalmologic surveillance in ETI-exposed infants [19]. Postpartum bilateral ureteroscopy with laser lithotripsy and nephrostomy tube removal was planned to eliminate the structural nidus for recurrent infection [3].

Multidisciplinary coordination and venous thromboembolism (VTE) prophylaxis

This case required simultaneous management by maternal-fetal medicine (MFM), critical care, pulmonology, urology, infectious diseases, interventional radiology (IR), hematology, nutrition, and anesthesia [5,8]. A key operational principle is designating a decision-making lead at each clinical phase while maintaining structured cross-specialty communication and a unified source-control philosophy. VTE prophylaxis with pneumatic compression devices was maintained during the ICU stay. Prophylactic-dose low-molecular-weight heparin (LMWH) was added thereafter, given the markedly elevated thrombotic risk associated with CF, pregnancy, and sepsis combined (aOR 3.59, 95% CI 1.33-9.69 for VTE in CF pregnancy) [20]. Shared decision-making regarding delivery timing, goals of care, and postpartum planning was documented throughout the antepartum course.

## Conclusions

This case reports a successful maternal and neonatal outcome in the setting of recurrent enterococcal urosepsis from indwelling bilateral nephrostomy tubes in a pregnant woman with cystic fibrosis (CF) maintained on CFTR modulator therapy. Despite four hospitalizations, significant hemodynamic compromise, acute anemia requiring transfusion, and fetal cardiac arrhythmia during the antepartum course, the patient delivered a healthy term male infant via spontaneous vaginal delivery with normal Apgar scores. Survival and fetal preservation depended on four key management principles: continuation of CFTR modulator therapy throughout critical illness; conservative fluid resuscitation given the vulnerable CF pulmonary physiology; deferral of elective nephrostomy tube manipulation during active sepsis followed by a structured four-week exchange schedule; and coordinated multidisciplinary care with clear phase-specific leadership.

Several teaching points warrant emphasis. The carbapenem-to-enterococcal microbiologic shift should be anticipated in patients receiving repeated broad-spectrum antibiotic courses, and long-term suppression strategies should account for both organisms in the colonization profile. A normal serum lactate does not exclude severe sepsis in patients with augmented renal clearance physiology; procalcitonin and the full clinical picture must guide management. The pharmacokinetic double alteration of CF and pregnancy represents an unresolved clinical and research priority, and therapeutic drug monitoring should be considered in future admissions. The healthy neonatal outcome and exclusive breastfeeding on ETI in this case contribute to the growing safety literature supporting continuation of CFTR modulator therapy throughout pregnancy and the postpartum period. As with all single-case reports, findings are hypothesis-generating rather than definitive; the retrospective design and absence of certain data points (arterial blood gas, fibrinogen, and D-dimer) represent limitations that should be considered when applying these observations to clinical practice.
